# Errors in perioperative antimicrobial use for hospitalized surgical patients

**DOI:** 10.1017/ash.2022.255

**Published:** 2022-09-30

**Authors:** Noah H. Boton, Payal K. Patel, Ronald E. Kendall, Cheryl Hershey, Mary Jarzebowski

**Affiliations:** 1 Division of Hospital Medicine, Department of Internal Medicine, Michigan Medicine, Ann Arbor, Michigan; 2 Ann Arbor Veterans’ Affairs (VA) Medical Center, Ann Arbor, Michigan; 3 Division of Infectious Diseases, Department of Internal Medicine, Michigan Medicine, Ann Arbor, Michigan; 4 Department of Anesthesiology, Michigan Medicine, Ann Arbor, Michigan

Hospitalized patients with severe infections may warrant intravenous (IV) antibiotics and surgical intervention for effective treatment of their infection. Surgical interventions require multiple transitions of care, which are well-studied sources of medication-related errors and inappropriate antimicrobial use.^1^ Transitions of care also exist within the operating room and contribute to an increased risk of adverse events.^2^

Errors in antibiotic use in the perioperative period may negatively affect patient outcomes. Patients who experience diminished serum and tissue concentrations of antibiotics during surgical closure have higher risks of postoperative wound infections.^3^ Furthermore, intraoperative bacteremia is common during elective surgeries.^4^ For patients requiring surgery for infection source control, maintaining adequate serum antibiotic levels may be particularly important in preventing bacteremia while infected tissue is surgically manipulated.

Although data exist on optimal timing of antibiotic administration for surgical antimicrobial prophylaxis,^5^ no studies have investigated perioperative antibiotic use for inpatients already on IV antibiotic regimens. The lack of guidelines introduces further complexity because it allows for additional and potentially inappropriate antibiotic administration for patients already receiving broad-spectrum antibiotics. In this study, we examined the incidence and nature of inappropriate antibiotic use in the perioperative period among inpatients on IV antibiotic regimens.

## Methods

We conducted a retrospective cross-sectional study at a Veterans’ Affairs medical center. We included all inpatients who underwent noncardiac surgery in 2019 who were aged >18 years or older and were on an IV antibacterial regimen prior to surgery. Patients receiving only surgical antimicrobial prophylaxis were excluded. Study and waiver of consent were approved by the Institutional Review Board of the VA Ann Arbor Healthcare System.

Through manual chart review by 2 physicians, we collected information on prescribed IV antibiotic regimens and timing of antibiotic doses in the perioperative period. Errors were classified as follows: missed doses, delayed doses, additional doses of prescribed IV antibiotics, or additional surgical antimicrobial prophylaxis. Delayed antibiotics were administered between 2 and 4 hours after the scheduled administration time, and missed antibiotics were either omitted or administered at least 4 hours after the scheduled administration time. Administration of surgical antimicrobial prophylaxis was defined as an error in patients already receiving IV antibiotics with the same or broader antimicrobial coverage.

## Results

**Fig. 1.**
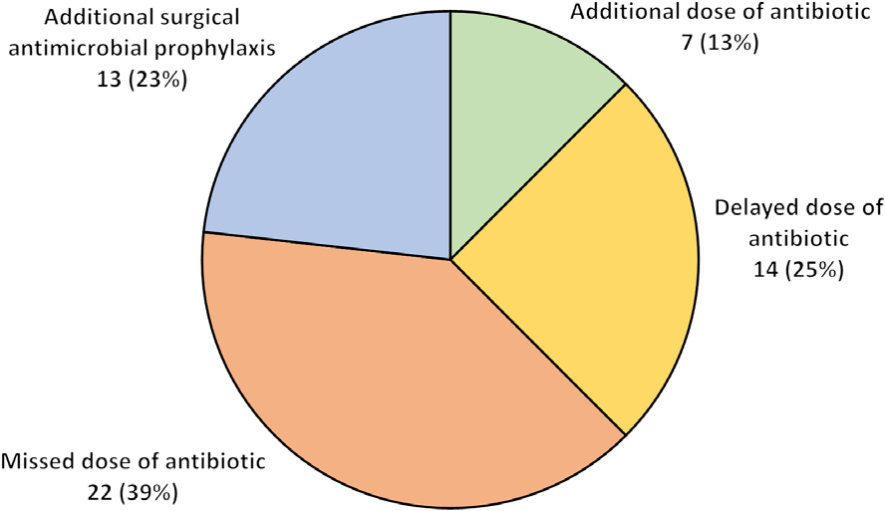
Errors in antibiotic administration in the perioperative period for inpatients on intravenous antibiotic regimens. Percent (%) indicates number of errors per total errors.

Of the 290 inpatients who underwent surgery in 2019, 163 patients (56%) received an IV antibiotic regimen prior to surgery. Complete data were available for 153 patients (94%). Errors in antibiotic administration in the perioperative period were identified in 60 patients (39%). The most common error was a missed dose of an IV antibiotic, which occurred in 22 patients (39% of errors) (Fig. 1). Delayed doses of IV antibiotics occurred in 14 patients (25% of errors). Administration of additional surgical antimicrobial prophylaxis occurred in 13 patients (23% of errors), and administration of an additional dose of the prescribed IV antibiotic occurred in 7 patients (13% of errors). Also, 4 patients experienced >1 type of error.

## Discussion

The appropriate use of surgical antimicrobial prophylaxis reduces the risk of surgical site infection,^5^ and national initiatives have been implemented to reduce errors in perioperative antibiotic administration.^6^ However, inpatients already on IV antibiotic regimens at the time of surgery may have been missed. Antibiotic misuse in these patients may compromise serum drug concentrations during manipulation of infected tissue, a critical point in infection control. Furthermore, the lack of guidelines for perioperative management in these patients may lead to unnecessary and inappropriate antibiotic use.

To our knowledge, this study is the first to evaluate the incidence and nature of errors in perioperative antibiotic administration for inpatients already on IV antibiotic regimens. We found these errors to be common; 39% of patients experienced at least 1 type of error related to antibiotic overuse, misuse, and underuse. The most frequent error (39% of errors) was a missed dose of an antibiotic. Also, antibiotic overuse (including both additional doses of prescribed antibiotics and additional surgical antimicrobial prophylaxis) accounted for 36% of errors. For example, patients already on broad-spectrum antibiotics for their infection were given additional antibiotics (eg, cefazolin) for surgical site infection prophylaxis. We suspect that these errors are related to the multiple transitions of care in the perioperative period. Some errors are also likely related to documentation of medications administered during an anesthetic in a separate part of the electronic medical record than medications administered on the hospital ward.

This study had several limitations. This was a single-center, retrospective study, and including multiple VA institutions would be a powerful next step. Second, the generalizability of these results may be limited because institutions using other electronic medical records may have different processes for documentation. However, we believe that the presurgical period in a patient’s hospitalization represents an underrecognized opportunity for antimicrobial stewardship given the transitions of care involved.

In conclusion, surgical patients account for significant proportions of inpatient antibiotic use,^7^ and misuse of antibiotics is common in these patients.^8^ Education-based stewardship interventions and clinical decision support tools implemented in surgical units have been shown to lead to reductions in antimicrobial consumption and improved trends in percentages of resistant bacterial strains.^9,10^ However, research evaluating interventions that specifically target perioperative antibiotic misuse for inpatients already on antibiotics for active infections is limited. This period may present a valuable opportunity for multidisciplinary stewardship intervention. Collaboration between antimicrobial stewardship programs, anesthesiology, and surgery departments should be encouraged to improve local practices in perioperative antibiotic prescribing and to address systems-based challenges that lead to inappropriate antibiotic use. Further research will be essential to the evaluation of outcomes of patients who experience errors in perioperative antibiotic administration.
